# Effect of silica nano-spheres on adhesion of oral bacteria and human fibroblasts

**DOI:** 10.1080/26415275.2020.1816175

**Published:** 2020-09-15

**Authors:** Pawel Kallas, Hua Kang, Håkon Valen, Håvard Jostein Haugen, Martin Andersson, Mats Hulander

**Affiliations:** a Department of Biomaterials, Institute of Clinical Dentistry, University of Oslo, Oslo, Norway; b Department of Chemistry and Chemical Engineering, Chalmers University of Technology, Gothenburg, Sweden; c Nordic Institute of Dental Materials, Oslo, Norway

**Keywords:** Nanopattern surfaces, bacterial attachment, oral bacteria, human fibroblasts, SiO_2_ nanoparticles, nano-patterning nanotopography anti-bacterial, early colonizer

## Abstract

**Objective:**

This study investigated the effect of surface nano-patterning on adhesion of an oral early commensal colonizer, *Streptococcus mitis* and the opportunistic pathogen *Staphylococcus aureus* and human fibroblasts (HDFa) in a laminar flow cell.

**Methods:**

Nanostructured surfaces were made by functionalizing glass substrates with 40 nm SiO_2_ nanoparticles. Gradients in nanoparticle surface coverage were fabricated to study the effect of nanoparticle spacing within a single experiment. Bacterial adhesion was investigated after 5 min of contact time by subjecting surfaces to a flow in a laminar flow cell. In addition, to examine the particles effect on human cells, the establishment of focal adhesion and spreading of primary human dermal fibroblasts (HDFa) were investigated after 4 and 24 h.

**Results:**

Adhesion of both *S. aureus* and *S. mitis* decreased on surfaces functionalized with nanoparticles and coincided with higher nanoparticle surface coverage on the surface. Both strains were tested on three separate surfaces. The regression analysis showed that *S. mitis* was influenced more by surface modification than *S. aureus*. The establishment of focal adhesions in HDFa cells was delayed on the nanostructured part of the surfaces after both 4 and 24 h of culturing.

**Significance:**

In the current manuscript, we have used a flow cell to investigate the effect of nanotopographies on *S. aureus* and *S. mitis* adhesion. The present findings are of relevance for design of future implant and prostheses surfaces in order to reduce adhesion of bacteria.

## 1. Introduction

Current literature has reported that more than 700 prokaryote species may inhabit the human oral cavity. Dysbiosis of the oral microbiome and changes in the proportion of species is associated with the development biofilm-associated diseases of the oral cavity such as, periodontitis, peri-implantitis and caries all if left untreated may lead to tooth loss [1]. A common treatment for replacement of lost or missing teeth is the placement of dental implants. On the surface of these materials, as on natural tooth, bacteria may adhere and form biofilms. The dental implant surfaces are engineered for bone cell attachment and osseointegration [2–5], however, this may also enhance bacterial adhesion and biofilm formation [6,7].

A biofilm is a biological community consisting of bacteria and a layer of organic and inorganic substances produced by these organisms [8]. Its formation on implant surfaces may lead to infection and breakdown of the implant supporting tissue [9,10]. The infection commences from the initial attachment of bacteria onto the implant surface followed by colonization and biofilm formation as previously described by Busscher et al. [11]. In the oral environment, both on teeth and dental implants, early colonizers (mainly oral streptococci) attach to the surface in the first place, initiating formation of biofilm [12–15]. Other microorganisms attach themselves to the extracellular polymeric substance (EPS) matrix in the biofilm or to already adhered bacteria. It has been shown that bacterial colonization of trans-mucosal implants occurs within 30 min after placement [16,17]. The establishment of a biofilm makes dental implant surfaces prone to infections and biomaterials associated infections (BAI) have been shown to be one of the leading causes of implant failure [18]. Microorganisms that grow in biofilm, compared to planktonic, free-floating cells, are much less sensitive towards different types of antibacterial treatments. Bacterial cells, which are an integral part of the biofilm, are characterized by a much higher resistance to conventional antibiotics compared to planktonic bacteria [19–21]. Additionally, the extracellular polymeric substance acts as a physical barrier that protects the bacteria from the host’s immune system [22].

The species associated with peri-implantitis have been shown to be the same core species as those associated with periodontitis [23]. However, the peri-implant microbiota has been reported in some instances to deviate from the periodontitis associated microbiota, with high numbers of staphylococci [24–26]. Clinical studies have reported high numbers of *S. aureus* in deep peri-implant pockets with suppuration and bleeding [27,28], and *S. aureus* is also associated with therapy resistant periodontitis [29–32]. The oral commensal ubiquitous colonizer *Streptococcus mitis,* has also been reported in higher numbers at implant sites with peri-implantitis, a finding also reported for periodontitis [26,33]. The essence of controlling or even preventing bacterial cells attachment is to understand the cell–material interactions during the process of bacterial adhesion [34].

Due to the occurrence of increased antibiotic resistance observed in general, and the reported frequent finding of submucosal antibiotic resistance bacteria from human peri-implantitis microbiota [35], development of new therapeutic and preventive strategies is called for. Modifying the dental abutment surface on the nano-scale level could be one of the approaches in the further use. Soft tissue integration on dental abutment separates the dental implant from the oral environment. This very barrier is often be impaired by biofilm formation, initiating inflammatory reactions in the peri-implant tissues. If such a biofilm migrates further to the endosseous part of the implant, the effect could lead to development of peri-implantitis and subsequent implant loss [36,37]. Previous studies have shown that the surface roughness and complex topography of the implant could have an effect on bacterial attachment [38,39], and that the presence of nanoparticles may exert an antimicrobial effect [40]. Metallic nanoparticles, such as gold (Au), silver (Ag) and zinc oxide (ZnO), are among these that show antimicrobial properties [41]. Silicon nanoparticles have also been shown to possess antibacterial [42] as well as biocompatible properties [43–45], and are hence promising candidates for use in dental applications.

One key to successfully limit the onset of BAIs is for eukaryotic cells to win the ‘race for the surface’, a term coined by Christina et al., that implicates that if eukaryotic cells have already established themselves on the surface of a biomaterial, chances increase that bacterial attachment is hampered and that the bacteria thus has lost the ‘race’ [46]. Mechanisms of eukaryotic cell adhesion have been studied extensively and apart from surface chemistry and mechanical properties of the substrate, surface nanotopography have also been found to play an important role in the adhesion and proliferation of several eukaryotic cell types [47]. Here we studied the establishment of focal adhesions (FA) in adult human dermal fibroblasts to investigate how the nanotopography of the surfaces affected initial cell adhesion.

Avoiding bacteria from adhering to the surface may prohibit the establishment of biofilm. The aforementioned scenario can be measured using a flow cell system that allows us to examine bacterial attachment under controlled conditions. Such systems have been already established and tested by different research groups [48–50]. To reduce the amount of experiments, materials that display a continuous change in properties or design along at least one specific direction (gradient) can be used. In our study, we used a flow cell system and gradients in surface coverage of nanoparticles to investigate whether different nanoscale topographies have influence on adhesion of bacteria to the surfaces.

## 2. Materials and methods

### 2.1. Materials

Sodium citrate tribasic dihydrate (Na_3_C_6_H_5_O_7_·2H_2_O, ACS reagent, ≥99.0%), citric acid (C_6_H_8_O_7_, ACS reagent, ≥99.5%), ammonium hydroxide solution (NH_4_OH, ACS reagent, 28.0–30.0% NH_3_ basis), 3-(ethoxydimethylsilyl)propylamine, and Tryptic soy broth (TSB) were purchased from Sigma-Aldrich, Oslo, Norway. Ethanol (C_2_H_5_OH, 96%) was purchased from Kemetyl, Oslo, Norway. Hydrogen peroxide (H_2_O_2_, 30%) was from VWR Chemicals, Oslo. Phosphate Buffered Saline (PBS) was purchased from by Lonza, Bornem, Belgium. Microscope glass slides was from Menzel-Gläser, Braunschweig, Germany. Silicon nanoparticles (SiO_2_, Levasil 100/45, 40 nm) were a gift from AkzoNobel, Angered, Sweden.

### 2.2. Preparation of nanostructured surfaces

Standard microscope glass slides were treated in an UV-O_3_ cleaner (BHK INC., Claremont, CA) for 15 min to remove organic contaminants and then washed in basic piranha solution (MQ water, NH_4_OH and 30% H_2_O_2_; 5:1:1) for 15 min at 80 °C. After that, surfaces where rinsed with MQ water and dried under N_2_ (g) flow. Immediately after, the surfaces were placed in a sealed container together with 3-(ethoxydimethylsilyl)propylamine in methanol (50/50; 200 µl each) in a watch glass for 30 min to amine-functionalize the substrates through evaporation of the silane onto the surfaces. Surfaces with homogenous distribution of nanoparticles were then prepared by submerging only half of the amine functionalized surface into a colloidal solution with 40 nm sized SiO_2_ nanoparticles (∼10 nM nanoparticle concentration) in 5 mM sodium citrate buffer (pH 4) for 15 min before thoroughly rinsing with MQ water and drying under a stream of N_2_ (g).

**Figure 1.**
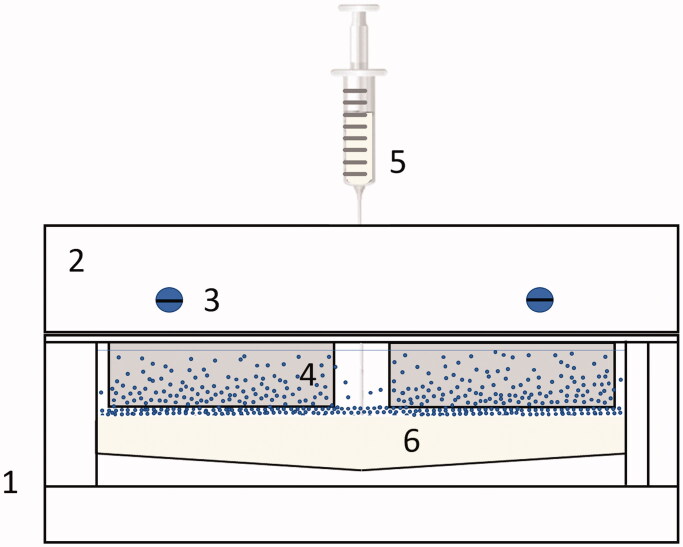
Gradient chamber (1): (2) Glass slide holder with mounting screws (3) to clamp glass slides (4), while buffer is injected at the bottom, below the nanoparticle solution using a syringe (5) into the chamber (6).

Surfaces with nanoparticle gradients where prepared using a modified version of an already established protocol [51]. Briefly, a solution of SiO_2_ nanoparticles (∼10 nM particle concentration in MQ water) was prepared and poured in a custom-built container (gradient chamber, Figure 1). After substrates were mounted in the holder and placed in the chamber, 50 mM citric buffer (pH 4.0) was injected in the bottom of the container using a syringe pump (2 ml/min) to obtain roughly 15 mm long gradients. Glass substrates were left in the nanoparticle solution for 90 min and then rinsed with MQ water and dried under N_2_ (g) flow.

Two different nano-structured surfaces were prepared and used throughout this work. One gradient surface, where the particle coverage varied smoothly across the surface, and one surface having two sides, a smooth part without particles and one with a surface coverage of 47%. The latter will be later referred as a bi-functional surface and was used for testing attachment of human dermal fibroblasts (HDFa) as well as a verification to *S. aureus* attachment.

### 2.3. Surface characterization

#### 2.3.1. SEM

A field emission scanning electron microscope (Hitachi S-4800, Tokyo, Japan) was used to characterize the experimental surfaces. A thin layer of platinum (3 nm) was sputtered onto surfaces using (Cressington 308 R Coating System, Cressington Scientific Instruments Inc., Watford, UK) prior to the SEM characterization. Pictures were taken at magnification of 50k and with the working distance (WD) set to 1.8 mm.

#### 2.3.2. Contact angle

Water contact angles measurements were performed on the experimental surfaces using NRL Contact Angle Goniometer Model 100-00-230 (Ramé-Hart, Inc. Mountain Lakes, NJ) to assess surface wettability. A small 5 µl MQ water droplet was applied on the homogenous surface, and the contact angles were measured at following time points: 30, 60, 120, 180, 240, and 300 s. This procedure was repeated 7 times, and an average contact angle value was then calculated. The measurements were performed on the bi-functional surfaces.

#### 2.3.3. XPS

X-ray photoelectron spectroscopy (XPS) was performed to confirm the removal of the amine containing 3-(ethoxydimethylsilyl)propylamine used for nanoparticle immobilization and to ensure similar surface chemistries on the smooth and nanostructured parts of the sample. Analyses were performed on areas measuring 400 × 500 µm, probing to a depth of ∼5 nm, using a Versa Probe III Scanning XPS Microprobe (Physical Electronics Physical Electronics, Inc., Chanhassen, MN) equipped with a monochromatic Al Kα (1486.6 eV) X-ray source. All measurements were performed at an incident angle of 45°. The measurements were performed on the bi-functional surfaces.

### 2.4. Bacterial preparation and growth

Both *S. aureus* (Newman strain) and *S. mitis* (NCTC 12261) were grown in tryptic soy broth (TSB) medium overnight at 37 °C and 5% CO_2_ atmosphere in centrifuge tubes. The overnight culture was diluted 10 times and left to grow again in the same conditions for 2 h. After that, samples were centrifuged at 5000 rpm (2912 rcf) at 21 °C to obtain a pellet (Thermo Scientific™ Heraeus Multifuge X3FR Centrifuge, Waltham, MA). The supernatant was discarded, and the pellet was resuspended in PBS (OD_600_ = 0.6, Thermo Scientific™ Spectronic 200E, Waltham, MA) before use in the experiments. The average colony-forming unit (CFU) was measured by culturing bacteria overnight on agar plates.

### 2.5. Bacterial attachment

**Figure 2.**
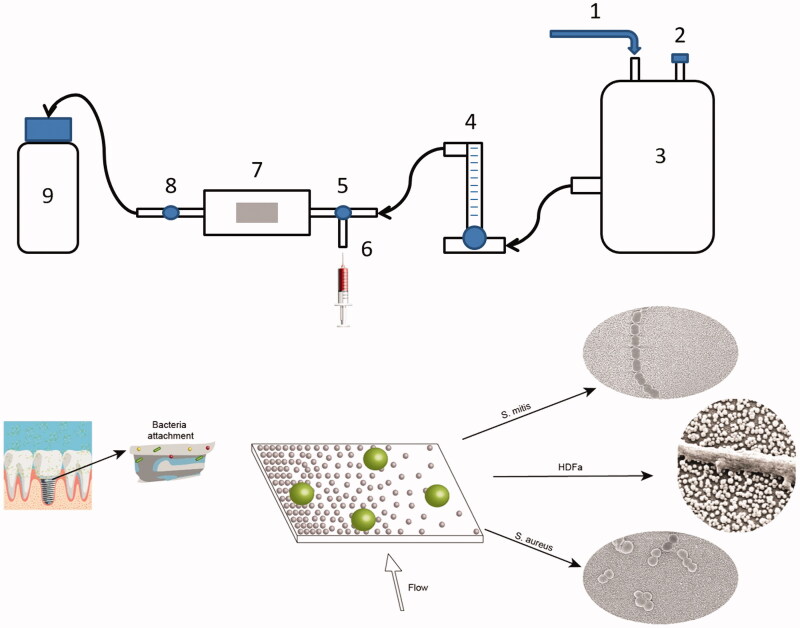
Flow chamber setup. 1: Pressurized air supply. 2: Air output. 3: Water tank. 4: Flow meter. 5: Right valve. 6: Syringe input. 7: Flow chamber with gradient sample (graphical example of the surface below). 8: Left valve. 9: Waste container. Arrows show direction of the flow. Implant image, reprint permission from Shutterstock illustrations-Number: 548568394/TrifonenkoIvan.

Experiments were performed in triplets. Prior to the experiments, the nanostructured surfaces were heat-treated in an oven at 400 °C in ambient air for 1 h, to remove residuals of the amine containing particle-binding silane, as well as atmospheric organic contaminants. After that, the sample was placed in the flow chamber, the system was flushed with distilled water for about 1 min at constant flow of 20 ml/min to remove any air bubbles trapped in the system. Then, 10 ml of bacteria in PBS were injected in the system manually using a syringe. The valves were then closed, and bacteria were let to adhere under static conditions for 5 min at room temperature. This procedure was followed by manually injecting 10 ml of 0.01% acridine orange (AO) to stain the cells for later viewing with fluorescence microscopy. After 3 min staining, valves were open again and sample was flushed for 5 min with distilled water at the same flow rate as before (20 ml/min). Each strain was tested three times on separate surfaces. The setup is presented in Figure 2.

### 2.6. Human dermal fibroblasts (HDFa)

Human dermal fibroblasts (cat. No. C-013-5C) were purchased from Gibco (Invitrogen, Carlsbad, CA). Cells were thawed and cultured in cell culture medium 106 supplemented with low serum growth supplement (LSGS) (Thermofisher Scientific, Indianapolis, IN) and cultured at an initial concentration of 5 × 10^3^ cells/cm^2^ in a 5% CO_2_ environment at 37 °C. Cells were grown until confluent and used in the attachment studies after passage 3 and 4 at a seeding concentration of 3 × 10^3^ cells/cm^2^. Cells were left to grow on the experimental surfaces under the above conditions for 4 and 24 h in individual wells in 12 well tissue culture plates. Experiments were performed in duplicates on two different occasions.

### 2.7. Fluorescence microscopy

For the counting of bacteria on the surfaces, the closed flow chamber, including glass cover, was transferred to a fluorescence light microscope (Olympus BX51, Tokyo, Japan). Bacterial adhesion was examined using a 10× magnification objective with U-MNB2 filter (excitation BP 470 − 490 and emission LP 520), and images were taken every 1 mm along the gradient, in 9 rows along the surface. Samples were stored overnight in 2.5% glutaraldehyde buffered with 0.1 M Sørensen’s phosphate buffer, and afterwards rinsed with ethanol and PBS and saved for later SEM imaging.

Human dermal fibroblasts were stained using a commercial kit for cell nucleus, actin filament and focal adhesions using DAPI, TRITC conjugated phalloidin and secondary FITC conjugated anti-vinculin antibodies respectively, by following the manufacturer’s protocol (FAK 100, Merck Millipore, Darmstadt, Germany). Images were captured using a Zeiss AxioImager (Carl Zeiss, Jena, Germany) fluorescence light microscope using a 40× objective and filters with excitation/emission wavelengths as follows: DAPI: 365/445, TRITC: 545/625, and FITC: 470/525 on three separate channels. An average of 62 images were randomly acquired on each experimental surface and the total number of cells for each treatment (smooth or nano) and time point ranged between 64 and 240 depending on the cell number on the individual images.

### 2.8. Image analysis

Image analyses were performed using the free software ImageJ (NIH, Bethesda, MD). To examine the gradient surfaces and calculate the coverage of nanoparticles, each picture was set to 8-bit, as well as the level of threshold was set to obtain visible contrast between nanoparticles and surface, which were later measured using the ‘Analyze particles’ feature of the program.

To calculate the number of bacteria, we used an already established macro plugin [50] which crop the original image in order to avoid artefacts from vignetting during the automated counting.

Images of human dermal fibroblasts (HDFa) were processed in ImageJ to identify individual cells and to calculate the total number of pixels per cell (DAPI + FITC + TRITC) and number of pixels corresponding to focal adhesions (TRITC). The number of nuclei (DAPI) was counted using ImageJ and checked manually to discern individual cells. To correct for differences in cell size, the ratio between focal adhesion per cell and the total cell area was calculated (Figure 7).

### 2.9. Statistics

Bacterial experiments were performed in triplicates and statistical analyses were conducted with SPSS version 25 (IBM Corp., Armonk, NY). To qualitatively state if the bacterial attachment was affected by the presence of nanoparticles, a bivariate Spearman correlation study was performed on the data sets of the two bacterial species, respectively. The results were interpreted as follows: no correlation if |*r*| < 0.3, correlation if 0.3 < |*r*| < 0.5, and strong correlation if 0.5 < |*r*| ≤ 1 [52]. Linear regression studies were performed with confidence intervals at 95% level between all the data points along the gradient in order to see if there was a linear correlation between the number of particles and the number of bacteria. The unstandardized regression coefficient (B) shows the change of bacteria number per 1% of surface coverage. Outlier values were kept, while extreme values (higher than outliers) were removed from the data set. The comparison in attachment of *Staphylococcus aureus* to bi-functional surfaces was conducted with GraphPad Prism version 8.3.0 for Windows (GraphPad Software, San Diego, CA).

Experiments with HDFa cells were performed in duplicates on two different occasions and data from 1500 individual cells were processed. The ratio between the number of focal adhesions and cell area was calculated using the total averages from each group (see Figure 7(E)).

## 3. Results

### 3.1. Surface characterization

#### 3.1.1. SEM

**Figure 3.**
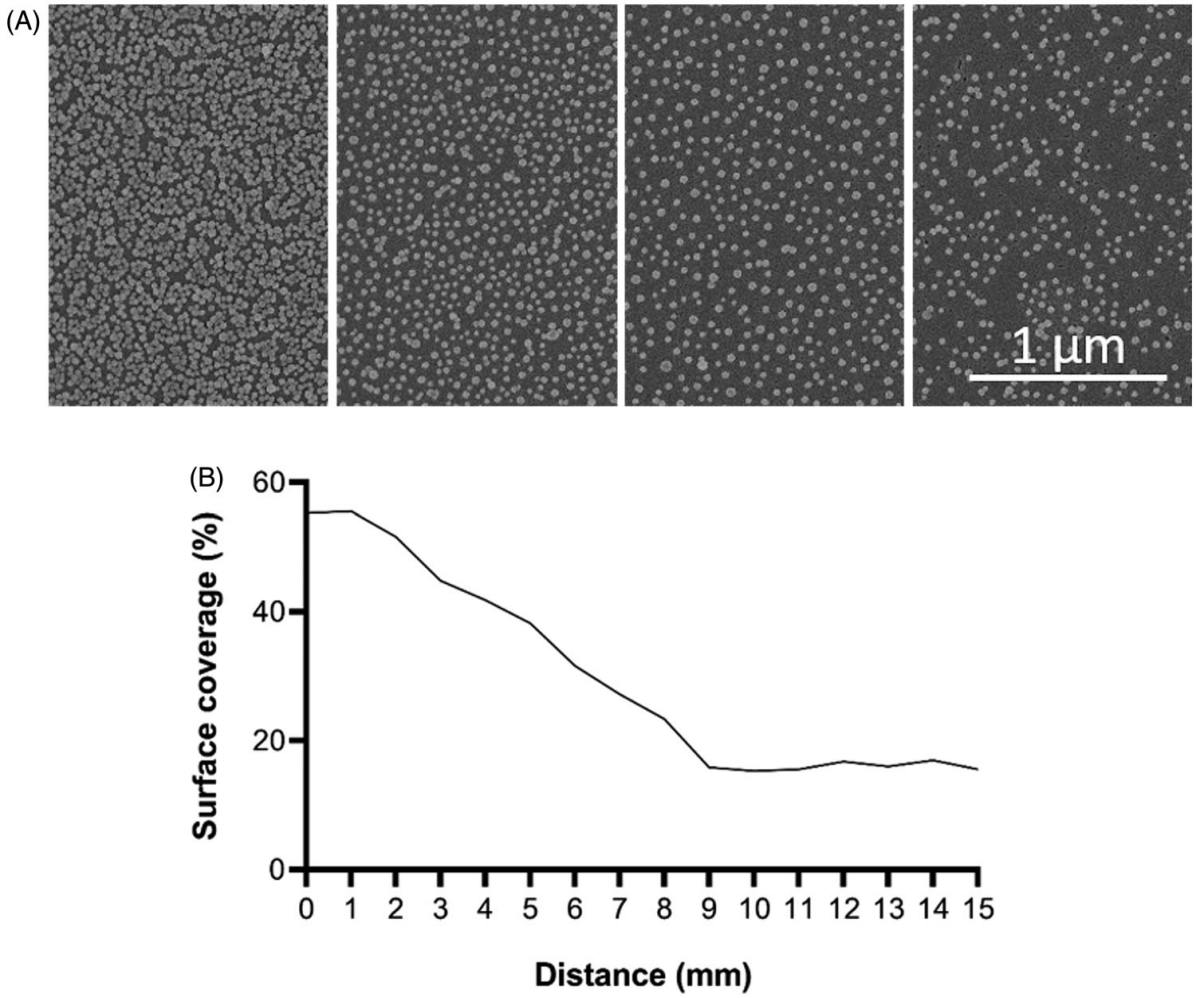
SEM images of nanoparticle gradient (A). Graph presenting surface coverage of nanoparticles (%) as a function of distance along the gradient (B).

The average diameter of the nanoparticles was 39 nm. The highest surface coverage varied between 35% and 58%. The number of nanoparticles and the area coverage of the nanoparticle coated surfaces used in the present study are shown in Figure 3.

#### 3.1.2. Water contact angle

The contact angle was significantly reduced on the homogenous nanoparticle coated surfaces compared to smooth surfaces both before and after heat treatment (Table 1). In addition, the contact angle was decreasing over time. All values were smaller than 90°, which means that surfaces were hydrophilic [53].

**Table 1.** Average contact angle values for nanostructured and smooth surfaces before and after heat treatment (*n* = 7, * *p* < 0.05: nano versus smooth).

Time (s)	Before heat treatment		After heat treatment	
	Nano	Smooth	Nano	Smooth
30	53* ± 1	69 ± 1	31* ± 2	43 ± 1
60	49* ± 1	66 ± 1	29* ± 2	41 ± 1
120	45* ± 1	63 ± 2	27* ± 2	39 ± 1
180	41* ± 1	58 ± 2	23* ± 2	35 ± 1
240	37* ± 1	54 ± 2	19* ± 2	31 ± 1
300	32* ± 1	50 ± 2	15* ± 2	27 ± 1

#### 3.1.3. XPS

From the XPS measurements, it was observed that the heat treatment effectively removed the nitrogen (N1s) containing 3-(ethoxydimethylsilyl)propylamine, which was used for the immobilization of the nanoparticles. The overall surface chemistry was approximately the same for the smooth and nanostructured part after the heat treatment. Analyses of the surface chemistry of the experimental surfaces are summarized in Table 2.

**Table 2.** Atomic percent of the most common species found in the experimental surfaces before and after heat treatment.

	Before heat treatment				After heat treatment			
	C1s	N1s	O1s	Si2p	C1s	N1s	O1s	Si2p
Nano	11	0.4	62	26	15	–	57	23
Smooth	18	1	59	20	17	–	55	22

Numbers represent % of the respective atomic species most commonly found on the surfaces before and after heat treatment.

### 3.2. Bacterial adhesion on nanoparticle gradients

**Figure 4.**
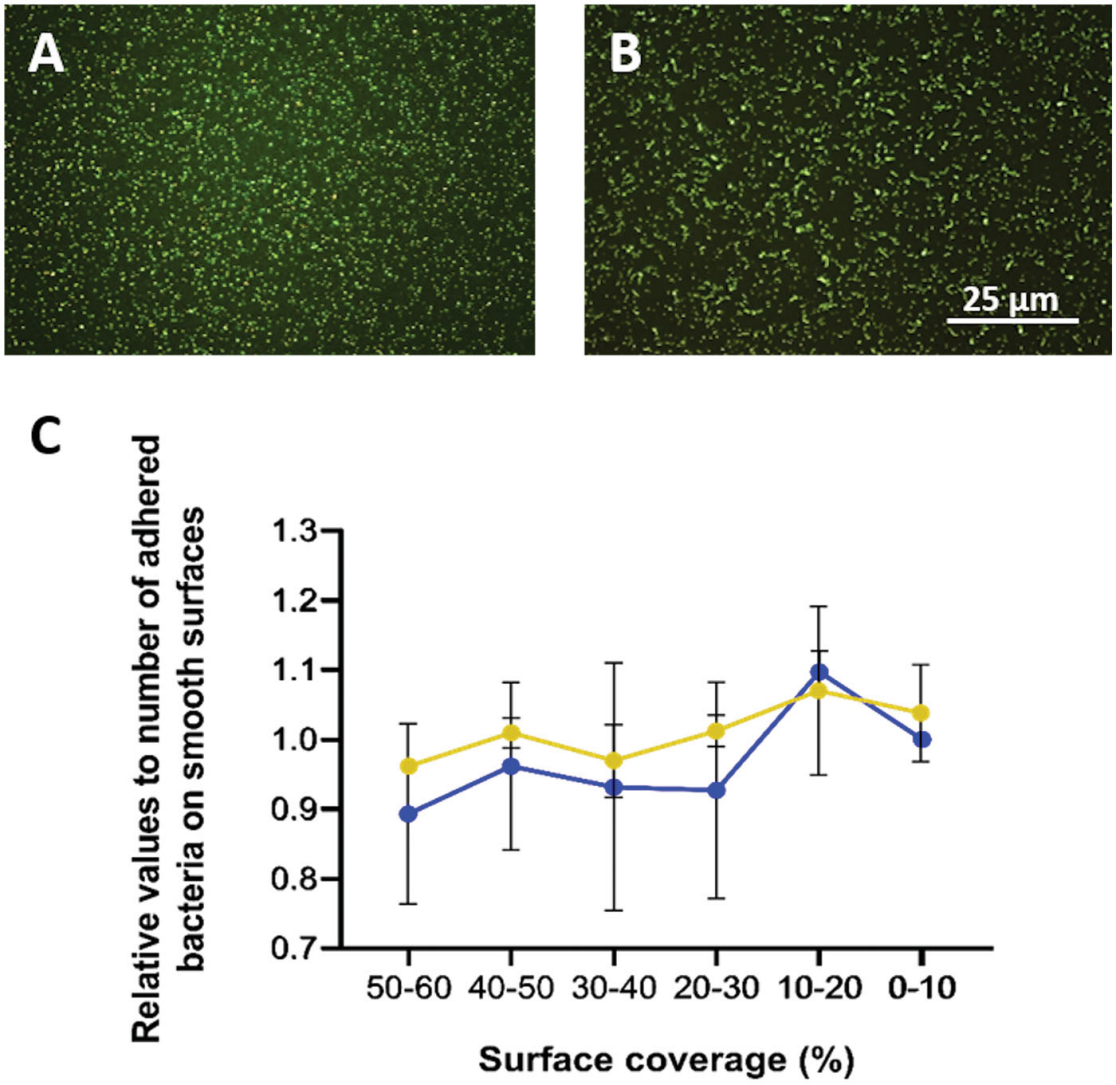
A: Fluorescence images (100× magnification) of *Staphylococcus aureus* and B: *Streptococcus mitis* after 5 min attachment and 3 min staining. C: Relative number of adhered cells of *S. aureus* (blue) and *S. mitis* (orange) with the standard error of the mean (SEM).

Relative numbers of adhered bacteria along the gradient for both types of bacterium are presented in Figure 4 where the *x*-axis represents the gradual change of surface coverage along the sample. The average colony-forming unit (CFU) for *S. aureus* was 0.90 × 10^8^ CFU/ml, and for *S. mitis* 0.86 × 10^8^ CFU/ml. Different adhesion abilities were observed for the two species (Table 3). Each strain was tested on three separate surfaces. It was found that *r* values for both *S. aureus* (experiments A–C) and *S. mitis* (experiments D–F) had positive values, with average 0.2 and 0.3, respectively. This means that higher number of bacteria attached to the area with lower surface coverage. The same pattern was observed in the regression studies, where *S. aureus* and *S. mitis* had positive B values, with average of 15.0 and 16.5, respectively. It also showed that *S. mitis* was influenced more by surface modification than *S. aureus*. Individually, two out of three experiments for *S. mitis* were significant, while for *S. aureus* only one experiment was significant. The highest degree of difference between experiments was observed for *S. mitis* (Table 3, E and F).

**Table 3.** Average Spearman correlation coefficients (*r*) and unstandardized regression coefficient (B) related to the change of bacteria number per 1% of surface coverage values for each experiment (*p < 0.05).

Bacteria	Experiment	*N*	Correlation coefficient, *r*	Unstandardized regression coefficient (B)
*Staphylococcus aureus*	A	162	0.2*	15.0*
	B	153		
	C	162		
*Streptococcus mitis*	D	162	0.3*	16.5*
	E	162		
	F	153		

**Figure 5.**
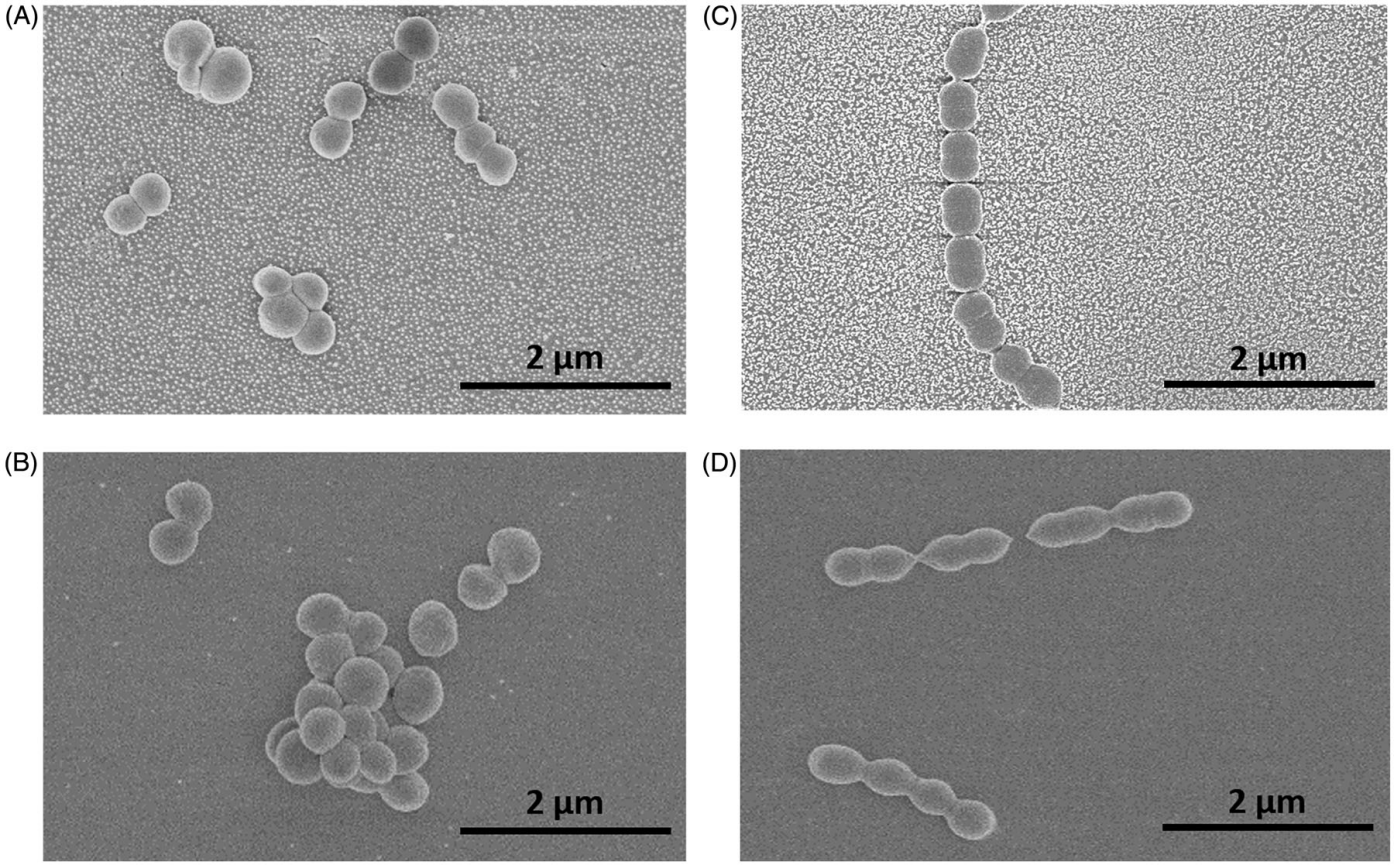
SEM images of bacteria on nanoparticle modified and smooth surfaces: *Staphylococcus aureus* (A – nano, B – smooth), *Streptococcus mitis* (C – nano, D – smooth).

Figure 5 shows representative images of *Staphylococcus aureus* (Figure 5(A,B)), and *Streptococcus mitis* adhered on the nanoparticle modified surfaces (Figure 5(C,D)).

### 3.3. Bacterial adhesion on nano versus smooth surface

**Figure 6.**
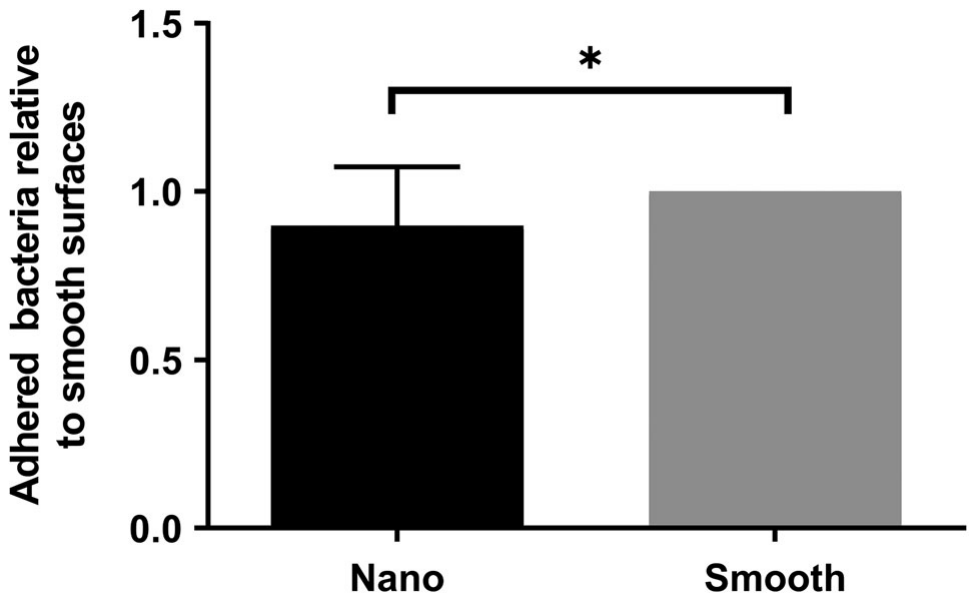
*Staphylococcus aureus* attachment to homogenous surfaces relative to high coverage of nanoparticles (Nano) in comparison to area with no nanoparticles (Smooth) with the standard error of the mean (SEM) (*p < 0.05).

To confirm the results observed for the gradient, homogenous surfaces with an average surface coverage of 47% was used. *S. aureus* showed a decreased adhesion to the surfaces with nano-particles compared to smooth surfaces, confirming the observations for the gradient (Figure 6).

### 3.4. Human dermal fibroblasts

**Figure 7.**
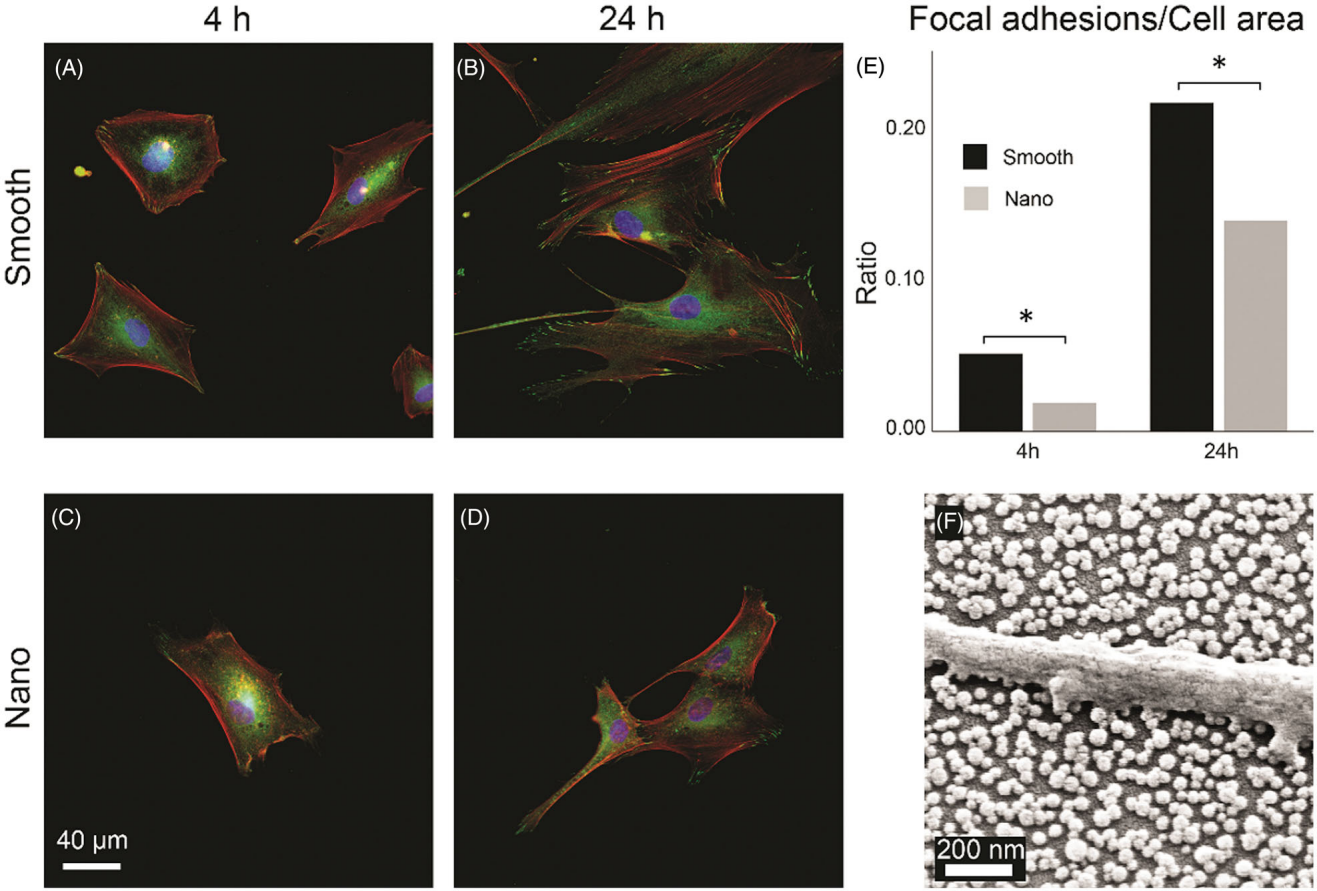
HDFa cells (A–D) stained for nucleus (blue), actin filaments (red), and focal adhesions (green) viewed at 400× magnification after culturing for 4 and 24 h on smooth and nanostructured surfaces. E: ratio between the number of pixels counted for focal adhesions and for the total cell area. (*p < 0.05). f: SEM image of HDFa cell filopodium resting on top of the SiO_2_ nanoparticles with no contact with the area in-between.

The effect of nanostructures on the development of focal adhesions (FA) in HDFa cells was assessed by quantifying the total number of focal adhesions per cell area of cells cultured on the bi-functional surfaces. In Figure 7(A–D), representative fluorescence microscopy images are shown for cells cultured for 4 and 24 h on nano or smooth areas of the bi-functionalized surfaces and stained for nucleus (blue), actin filaments (red) and focal adhesions (green). In Figure 7(E), a summary of the ratio between pixels from the green channel (FA) and the sum of pixels counted per cell is shown for all analyzed cells. Data are corrected for differences in cell sizes.

HDFa cells were examined using SEM to investigate the cell-substrate interaction. A general observation was that cells on the nanostructured part of the surfaces was seen resting on top of the nanoparticles with no contact with the surface in-between particles. An example of a HDFa cell cultured for 24 h is shown in Figure 7(F) where a part of a filopodium protruding from a cell is seen resting on top of the nanoparticles.

## 4. Discussion

The purposes of our experiments were to investigate whether bacterial and human cell adhesion is influenced by nanoscale topographies. The motivation behind this choice of surface is the fact that dental abutment materials often exhibit topographical features at the nano-scale [54]. However, from the current literature, it is unclear what role nanostructured surfaces have on adhesion of bacteria and subsequently biofilm formation and biomaterial associated infections (BAI) [55–58]. Therefore, our study aimed to systematically investigate bacterial adhesion to well-defined nanostructured substrates. It was previously shown that gradients of nanoparticles on surfaces may be used to systematically study bacteria attachment as a function of nanotopography under flow conditions [50]. The present study used the same concept focused on two different types of bacterium and their ability to attach to nanostructured surfaces. These choice of bacteria (*Staphylococcus aureus* and *Streptococcus mitis*) take part in dental implant’s surface colonization and have been shown to lead to BAI [26,59].

We tested 5 min bacterial attachment on standard microscope glass slides, and SiO_2_ nanoparticles were chosen as a coating material due to their low toxicity, biocompatibility and chemical inertness [60]. The reason why we used short exposure time was that our test system is a model system to investigate role of nanotopography on bacterial adhesion avoiding interference from division of adhered bacteria. In addition, it has previously been observed that transition from reversible to irreversible adhesion is a rapid process [61]. To detect difference in the current flow system, short incubation time was used to find ranges of nanotopographies which could be useful for future abutment surfaces. Once such a regime of nanotopographies has been established, abutment surfaces with longer incubation times need to be performed to validate clinical relevance. Our findings show that nanoparticles increased the wettability of the surface, even though XPS shows that the chemistry is roughly identical. This shows that the difference in wettability was linked to the surface topography. In addition, a decrease in contact angle was observed after thermal treatment of the surfaces, confirming the removal of 3-(ethoxydimethylsilyl)propylamine not involved in immobilization of the nano-particles. This has been also reported by Brink et al. [62], where contact angle decreased significantly after thermal annealing for 10 min at a temperature of about 120 °C. In comparison to our study, their surfaces were heat-treated at a lower temperature and time as well as on a hot plate instead of in an oven.

Investigation of bacterial adhesion showed that both *S. mitis* and *S. aureus* adhered less strongly to surfaces functionalized with SiO_2_ nanoparticles and confirm previous findings where surface nanotopography has been found to decrease the ability of *S. epidermidis* to attach [50]. Puckett et al. [63] tested *S. aureus* and *S. epidermidis* towards their ability to attach onto nanostructured titanium surfaces. They found that nanostructured surfaces decrease the adherence of all tested bacteria, comparing to smooth surfaces. Another study performed by Caous et al. [64], examined how *S. mitis* reacts towards machined (relatively smooth) and anodized (rough) titanium surfaces. Their results showed that *S. mitis* is more likely to attach to a smoother surface, rather than a rough one. Adherence of *S. mutans* and *S. sanguinis* to nanotextured titanium surfaces by Narendrakumar et al. [65] increased with increase of nanotubule diameter.

Nanoparticle modified surfaces have also been shown to affect biofilm formation. Applerot et al. [66] deposited zinc oxide (ZnO) nanoparticles on a glass slide, and tested biofilm formation ability of *Escherichia coli* and *S. aureus*, which resulted in antibiofilm activity of the coated film. In another study by Lellouche et al. [67], *E. coli* and *S. aureus* were used to compare biofilm formation on catheters coated with magnesium fluoride (MgF_2_) nanoparticles to uncoated controls. For a period of 1 week, they showed that the presence of MgF_2_ nanoparticles significantly reduced bacterial adhesion.

From our FLM images of HDFa cells, it was evident that the morphology of the cells was affected by the presence of nanoparticles. Cells cultured on the nanostructured part of the bi-functional surfaces were generally found to be more elongated and exhibited less spreading and gave the impression of being less ‘mature’ or established than cells on the smooth part (Figure 7(A–D)). When calculated, the number of focal adhesions per cell area was also found to be lower in the nano group (Figure 7(E)). In the SEM image in Figure 7(F), a part of a filopodium protruding from a HDFa cell cultured for 24 h on the nanostructured part is shown. The filopodium is seen resting on top of the particles with no contact with the surface in-between and hence the only contact point with the substrate is governed by the 40 nm sized nanoparticles. This could indeed explain the lower ratio of focal adhesions expressed in cells cultured on the nanostructured part. In a previous study where SiO_2_ nanoparticles in the same size range were used, cell-type-specific response where some cell types expressed higher number of focal adhesions on nanostructured surfaces while the opposite was found for others [68]. This indicate that for eukaryotic cells, fine-tuning of the biomaterial surface for certain cell types, at the nanoscale, could impact the outcome of the ‘race for the surface’ and thus be an important feature for preventing BAIs.

Unravelling and understanding the details of bacterial and host cell adhesion on nanostructured surfaces could potentially lead to new approaches in the design of implant surfaces, thereby preventing bacterial attachment, and as a result – lowering the risk of biomaterials associated infections.

## 5. Conclusions

We have investigated the early adhesion of *S. aureus*, *S. mitis* and the attachment of primary human dermal fibroblasts on smooth and SiO_2_ nanopatterned surfaces displaying the same surface chemistry. For both *S. aureus* and *S. mitis,* adhesion was decreased on nanostructured compared to smooth surfaces and correlated with surface coverage. For human dermal fibroblasts we found a reduced number of focal adhesions for cells cultured on the nanostructured part of the bi-functional surfaces for both 4 and 24 h. We attribute these findings to the limited contact points between the cells and the substrate provided by the nanoparticles.
